# Dioxin-like compounds are not associated with bone strength measured by ultrasonography in Inuit women from Nunavik (Canada): results of a cross-sectional study

**DOI:** 10.3402/ijch.v72i0.20843

**Published:** 2013-05-29

**Authors:** Alexandra-Cristina Paunescu, Pierre Ayotte, Éric Dewailly, Sylvie Dodin

**Affiliations:** 1 Axe santé publique et pratiques optimales en santé, Centre de recherche du CHU de Québec, Québec, Canada; 2 Laboratoire de toxicologie, Institut national de santé publique du Québec (INSPQ), Québec, Canada

**Keywords:** bone strength, stiffness index, dioxin-like compounds, polychlorinated biphenyls, Inuit women

## Abstract

**Background:**

Bone strength in Inuit people appears lower than that of non-Aboriginals. Inuit are exposed to persistent organic pollutants including dioxin-like compounds (DLCs) through their traditional diet that comprises predatory fish and marine mammal fat. Results from experimental and population studies suggest that some DLCs can alter bone metabolism and increase bone fragility.

**Objective:**

This cross-sectional descriptive study was conducted to examine the relationship between the stiffness index (SI) and plasma concentrations of total DLCs or specific dioxin-like polychlorinated biphenyls (DL-PCBs) in Inuit women of Nunavik (Northern Quebec, Canada).

**Methods:**

SI was determined by ultrasonography at the right calcaneus of 194 Inuit women aged 35–72 years who participated to *Qanuippitaa?* How Are We? Nunavik Inuit Health Survey in 2004. Plasma total DLC levels were quantified by measuring the aryl hydrocarbon receptor–mediated transcriptional activity elicited by plasma sample extracts in a cell-based reporter gene assay. Plasma concentrations of DL-PCBs nos. 105, 118, 156, 157, 167 and 189 were measured by gas chromatography–mass spectrometry. We used multiple linear regression analyses to investigate relations between total DLCs or specific DL-PCBs and SI, taking into consideration several potential confounders.

**Results:**

Neither total plasma DLCs nor specific DL-PCBs were associated with SI after adjustment for several confounders and covariates.

**Conclusion:**

Our results do not support a relation between exposure to DLCs and bone strength measured by ultrasonography in Inuit women of Nunavik.

Inuit people live in extreme climatic conditions and exhibit unique dietary and lifestyle habits. They are exposed to persistent environmental contaminants such as polychlorinated biphenyls (PCBs) and methylmercury through their traditional diet that comprises large amounts of marine mammal meat and fat (1, 2).

Studies conducted during the 1970s and early 1980s have reported an earlier onset and a greater age-related bone loss in Inuit compared to US Caucasians (3–6). Cortical bone thickness in Inuit was also reported to be smaller than in Caucasians (5–8). A decreasing trend in cortical bone thickness was noted from Mongoloid groups of Umnak-Kodiak on the Bering Sea going eastward to Greenland, with a demarcation point between Inupiaq and Yupik Inuit (9). Histological analyses at the femur revealed that the Inuit have more osteons per unit area than US Caucasians, although osteons are similar in size (6). Some authors have suggested that Inuit differ from other populations with regard to the osteon remodelling pattern (8, 10–12). Côté et al. (13) performed quantitative ultrasound (QUS) measurements at the right calcaneus of Greenlandic Inuit women and reported that their mean stiffness index (SI) value was 9.5% lower than that of southern Quebec Caucasian women (13).

It was hypothesized that the osteological features of Inuit could be related to their diet rich in animal proteins, phosphorus and nitrogen and low in calcium (3, 4, 14), or to genetic factors, sun exposure and cultural factors (15). Smoking, chronic use of oral steroids and the low dietary calcium intake were identified as the main risk factors for low bone mineral density (BMD) measured at the calcaneus in Alaska Native women (16).

In the study by Côté et al. (13), plasma concentrations of PCB-156, a mono-ortho substituted PCB congener displaying some affinity for the aryl hydrocarbon receptor (AhR), were negatively associated with calcaneal QUS parameters in Greenlandic Inuit women (13). Environmental chemicals that can bind the AhR are referred to as dioxin-like compounds (DLCs). DLCs are lipophilic and persistent compounds that are biomagnified in food chains; the main source of exposure to DLCs in human populations is the consumption of fat from contaminated animals (17). Inuit are exposed to DLCs mainly through their consumption of marine mammals as part of their traditional diet (2, 18). DLCs induce antioestrogenic activity in bone tissue (19) and therefore could be involved in BMD reduction. Results of *in vitro* studies (20) and experimental studies in rats suggest that 2,3,7,8-tetrachloro-dibenzo-*p*-dioxin (TCDD), the most potent DLC, affects bone metabolism and increases bone fragility (21, 22). The relation between exposure to DLCs and bone quality, whether assessed by BMD or QUS parameters, has seldom been investigated in humans. The few studies available focused on specific dioxin-like polychlorinated biphenyls (DL-PCBs) and did not consider total DLC exposure (13, 23, 24).

SI reflects the structural parameters and elastic properties of the calcaneus and provides complementary information to that of BMD on bone strength. Osteoporotic patients who are more at risk of fragility fracture generally exhibit lower QUS parameter values than healthy subjects (25).

The main objective of this study was to examine the relationship between total plasma concentrations of DLCs (measured using an AhR-responsive cell-based reporter gene assay) or specific DL-PCBs (measured by gas chromatography coupled to electron capture negative ion mass spectrometry) and SI measured by ultrasonography at the calcaneus of Inuit women from Nunavik.

## Methods

### Population

The Inuit Health Survey entitled “Qanuippitaa? How Are We?” was organized by the Nunavik Regional Board of Health and Social Services and took place from August 27 to October 1, 2004, in the 14 Inuit communities of Nunavik (located north of the parallel 55°N in Québec, Canada) (26).

The target population for the survey was the entire population living permanently in Nunavik (9,632 inhabitants according to the 2001 Canadian census), with the following exclusions: (a) households where there was no adult Inuit aged 18 years and above; and (b) residents of collective dwellings (houses, hotels, nursing homes, hospitals and prisons). The target population represented 91% of the total population of Nunavik (26). The survey plan was a complex 2-stage stratified random sampling. The first stage was the selection of a random sample of private Inuit households stratified by community, with proportional allocation according to the community size. In the second stage, all eligible individuals living in these households were asked to participate in the survey. Among the 677 eligible households, 521 agreed to participate in the survey, which represents a total weighted response rate of 77.8%. These 521 houses have generated 2,550 individuals (26).

Participants answered a series of questionnaires available in English and Inuktitut that were administered by the research staff. Those aged 18–74 years also participated in a clinical session, which involved collection of blood samples and anthropometric measurements. All women aged 35–74 years who responded to the household questionnaire were eligible for QUS measurement. Among the 317 eligible women, 207 (aged 35–72 years) underwent QUS measurement. The weighted mean proportion of participants to this measure was therefore 65.5% and the global weighted response rate was 51% (26). Among the 207 participants, 12 of them were excluded (3 were not Inuit, 8 had no plasma DLC concentration and 2, including 1 without DLC value, were pregnant at the time of the study). Therefore, 195 Inuit women completed the study.

The project was approved by the *Comité d’éthique de la recherche de l'Université Laval* and the *Comité d’éthique de santé publique du Québec*. Participation in the study was voluntary and each participant signed a consent form. All information regarding the participants was kept strictly confidential.

## Measurements and analyses

### Bone measurements

SI was measured at the right calcaneus of participants using the portable ultrasound instrument Achilles Insight (GE Healthcare Lunar, Madison, WI, USA) (27). SI value was calculated automatically by the instrument from 2 QUS parameters, the speed of sound (SOS, m/s) and the broadband ultrasound attenuation (BUA, dB/MHz), using the formula from the manufacturer: [SI%=(0.67*BUA)+(0.28*SOS)−420].

The inspection of the instrument membranes was performed daily before the first measurement of the day, followed by a quality control test. The instrument was calibrated daily using the bone-mimicking phantom provided by the manufacturer. The accuracy was assessed *in vitro* based on 36 repeated measurements conducted with the phantom of the manufacturer; the average coefficient of variation (CV) for SI was 0.15%.

### Anthropometric measures

Body weight (kg; balance beam scale) and height (cm) were determined by research nurses using standardized techniques.

### Laboratory analyses

Fasting blood samples were processed within 2 hours of collection. Samples collected in tubes containing EDTA as the anticoagulant were centrifuged, the plasma transferred into glass vials (Supelco) pre-washed with hexane and stored frozen at −20°C. Samples were shipped to the *Laboratoire de Toxicologie* of the *Institut National de Santé Publique du Québec* (INSPQ, Québec, Canada), where total DLCs and DL-PCB analyses were conducted.

The preparation of purified extracts for PCBs and AhR-mediated transcriptional activity analyses was previously described (28). An AhR-responsive cell-based reporter gene (luciferase) assay was used to measure the transcriptional activity elicited by plasma sample extracts. Total plasma DLC concentration was interpolated using a TCDD standard curve, and results were expressed in pg TCDD-equivalents (EQ)/L.

Data on the precision and relative accuracy of the AhR-responsive bioassay were obtained through the quantification of the NIST SRM (standard reference material) 1589a (National Institute of Standards and Technology, Gaithersburg, MD, USA) sample. The toxic equivalent concentration of this reference sample, calculated as the toxic equivalency factor-weighted sum of the certified (or reference) concentrations of PCDDs, PCDFs, non-ortho PCBs and mono-ortho PCBs was 189.1 pg/L. One NIST SRM 1589a per analytical batch (n=30) was analysed, and a mean transcriptional activity of 170.7 pg TCDD-EQ/L and a CV of 26% was obtained. The apparent bias of the reporter gene bioassay was −9.7%. The limit of detection (LOD) was 30 pg TCDD-EQ/L (28).

Purified extracts were analysed for 44 PCB congeners, including 6 DL-PCBs (nos. 105, 118, 156, 157, 167 and 189), by gas chromatography coupled to electron capture negative ion mass spectrometry (28, 29). The LOD for the DL-PCBs was 10 ng/L. Between-day CVs based on repeated measures of the NIST SRM 1589a ranged between 5.3 and 11.1% depending on the congener.

Concentrations of total cholesterol (TC) and total triglycerides (TGs) in plasma samples were determined by enzymatic methods at the *Centre de recherche sur les maladies lipidiques* (Centre de recherche CHU de Quebec, QC, Canada). The total plasma lipid (TL) concentration was calculated from TC and TG concentrations (all of them expressed as g/L) by using the following formula (30):TL=2.27TC+TG+0.623.

The fatty acid composition of phospholipids in erythrocyte membranes was also determined at the *Centre de recherche sur les maladies lipidiques* according to the method previously described (28). Polyunsaturated fatty acid (PUFA) content was expressed as the percentage of all fatty acids in membrane phospholipids. Total omega-3 PUFAs comprise the following fatty acids: α-linolenic acid (C18:3*n*–3), docosapentaenoic acid (C22:5*n*–3), docosahexaenoic acid (C22:6*n*–3), eicosapentaenoic acid (C20:5*n*–3), C18:4*n*–3, C20:3*n*–3 and C20:4*n*−3. Total omega-6 PUFAs refer to the sum of linoleic acid (C18:2*n*−6), arachidonic acid (C20:4*n*−6), C18:3*n*–6, C20:2*n*–6, C20:3*n*–6, C22:2*n*–6, C22:4*n*–6 and C22:5*n*–6.

Methods for quantifying blood levels of toxic metals (lead, mercury) as well as plasma retinol and vitamin D concentrations have been published elsewhere (26, 31).

### Questionnaires

Questionnaires were used to document socio-demographic variables (date and place of birth; level of education: none or primary/secondary and higher), lifestyle habits (smoking during the last year, yes/no) and gynaecological history (menopausal status, postmenopausal/non-menopausal; parity, yes/no). Women were considered postmenopausal if they had no menstrual period for 1 year before their recruitment in the study. Medical files were consulted to document the use of calcium and vitamin D supplements (yes/no).

### Statistical analyses

Descriptive statistics [mean, standard error (SE) of the mean, minimum, maximum for quantitative variables or numbers and percent per modality for categorical variables] were presented for our 195 participants.

DLC concentrations were below the LOD (30 pg TCDD-EQ/L) for 32 participants. In these cases, we imputed a value between 0 and the LOD selected by simple random sampling with replacement. Plasma concentrations of DL-PCBs nos. 105, 156, 157, 167 and 189 were below the LOD (10 ng/L) for 5, 3, 23, 19 and 68 participants, respectively. In these cases, we imputed a value equal to 5 ng/L (LOD/2).

Pearson's correlation coefficients were calculated between SI and plasma levels of total DLCs or individual DL-PCB congeners. The relationships between total DLCs or DL-PCBs and SI were examined by multiple linear regression analyses. Two multivariate models were proposed. The models were adjusted for multiple confounders and covariates, including the total concentration of plasma lipids.

Multicollinearity diagnostics were performed for all independent variables. For continuous variables, the normality, linearity and homoscedasticity of residuals were tested graphically and through hypothesis testing. Box–Cox procedures were used to resolve problems encountered with the hypotheses of normality and/or homoscedasticity in the multiple regression models for SI, which was subsequently log-transformed.

Populational weight was calculated by the *Institut de la Statistique du Québec* (ISQ) and used to obtain all estimates, while SEs were computed using the bootstrap procedure (26). ISQ considered that 500 sub-samples were sufficient to provide bootstrap weights for the survey.

A *p*-value <0.05 in the bilateral situation was considered statistically significant. Statistical analyses were performed with SAS version 9.2 software (SAS Institute Inc., Cary, NC, USA) and SUDAAN version 10.0 software.

## Results

### Characteristics of participants

The main characteristics of the participants are listed in Table I. Inuit women were aged between 35 and 72 years and were predominantly non-menopausal. A majority of participants had children, a low level of education and were smokers.

**Table I:** Characteristics of Inuit women from Nunavik

Characteristic	N^a^ (N^b^)	AM^c^ (SE)	Range	GM^d^ (SE)
SI (%)	1,140 (195)	78.7 (1.0)	39–135	
DLCs (pg TCDD-EQ/L)	1,140 (195)	120.4 (7.1)	0.7–570	74.0 (5.3)
DL-PCB 105 (ng/L)	1,140 (195)	115.0 (7.0)	5–620	69.2 (4.0)
DL-PCB 118 (ng/L)	1,140 (195)	582.7 (35.5)	22–3,000	352.1 (19.6)
DL-PCB 156 (ng/L)	1,140 (195)	163.3 (13.0)	5–1,800	98.5 (5.7)
DL-PCB 157 (ng/L)	1,140 (195)	60.4 (4.8)	5–520	35.1 (2.1)
DL-PCB 167 (ng/L)	1,140 (195)	55.2 (3.8)	5–460	33.7 (1.9)
DL-PCB 189 (ng/L)	1,140 (195)	25.6 (2.2)	5–270	15.3 (0.9)
ΣDL-PCBs^e^ (ng/L)	1,140 (195)	1,002 (59)	47–4818	633.8 (33.3)
Mercury (nmol/L)	1,140 (195)	132.1 (6.7)	4.9–760	94.5 (5.0)
Lead (µmol/L)	1,140 (195)	0.28 (0.01)	0.04–1.5	0.23 (0.01)
Age (years)	1,140 (195)	48.6 (0.5)	35–72	
Weight (kg)	1,124 (192)	66.6 (1.0)	37.6–118	
Height (cm)	1,140 (195)	152.3 (0.3)	142–164	
Omega-3 PUFAs (%)	1,140 (195)	11.9 (0.2)	1.7–19.5	
Omega-6 PUFAs (%)	1,140 (195)	23.3 (0.3)	12.6–32.4	
Retinol (µmol/L)	1,111 (191)	2.16 (0.04)	1.04–3.58	
Vitamin D (nmol/L)	1,134 (194)	30.1 (0.7)	5.9–67.5	
Total plasma lipids (g/L)	1,135 (194)	6.4 (0.1)	4.1–11.4	
	N^f^ (%)	N^b^		
Menopausal status	1,139.7	195		
Postmenopausal	486.4 (42.7)	79		
Non-menopausal	653.3 (57.3)	116		
Level of education	1,093.3	191		
None or primary school	590.7 (54.0)	103		
Secondary or higher	502.6 (46.0)	88		
Supplement use^g^	1,139.6	195		
Yes	115.4 (10.1)	19		
No	1,024.2 (89.9)	176		
Tobacco use	1,131.4	193		
Yes	827.3 (73.1)	144		
No	304.1 (26.9)	49		
Parity	1,123.4	192		
Yes	1,057.7 (94.1)	182		
No	65.7 (5.9)	10		

a Weighted and rounded size

b sample size

c arithmetic mean

d geometric mean

e sum of DL-PCB nos. 105, 118, 156, 157, 167 and 189

f weighted size

g calcium and vitamin D supplements in the last 12 months.

Only 10% had taken calcium and vitamin D supplements during the last 12 months. Forty-four percent had a plasma vitamin D level considered as critical (<27.5 nmol/L) (32); 74% displayed inadequate levels (<37.5 nmol/L), whereas 94% showed levels considered to be minimal (<50 nmol/L) (33). Plasma concentrations of retinol (vitamin A) were normal in 70% of Inuit women (300–700 mg/L, 1.05–2.44 mmol/L) (34); <1% had a level below the normal range and 30% were above.

With regard to blood mercury levels, 98% of Inuit women exceeded the maximum blood mercury level of 16 nmol/L observed in women from the Quebec City area (35), whereas 50% exceeded Canada's alert level of 99.7 nmol/L (36). The maximum blood lead level of 0.32 µmol/L noted in women from the Quebec City area (35) was exceeded by 32% of participants, while 11% exceeded Canada's alert level of 0.48 µmol/L (37).

### Pearson's correlation coefficients (r)

SI values (log-transformed) were negatively correlated with total DLCs, DL-PCBs and age (r=−0.34 to −0.58, p<0.0001). Total DLCs and the 6 DL-PCBs were intercorrelated (r=0.58–0.69, p<0.0001); all of them were positively correlated with age (r=0.58–0.75, p<0.0001).

### Results from multivariate analyses

#### Main exposure variables

In multivariate models adjusted for a larger number of confounding factors and covariates (model 1; Table II), the significant associations between DL-PCB nos. 156, 157, 167 and 189 and SI (log) observed in models adjusted only for age and TLs (model 2) were no longer significant.

**Table II:** Results of explanatory multiple linear regression analyses: SI (log) models

Main exposure variables	Model 1^a^ N^c^=1,041 (N^d^=180)			Model 2^b^ N^c^=1,135 (N^d^=194)		
	
Contaminant	R^2^	Regression coefficient (SE)^e^	p-Value^f^	R^2^	Regression coefficient (SE)^e^	p-Value^f^
DLCs (pg TCDD-EQ/L)	0.4184	1.6468 (1.3199)	0.2128	0.3303	−0.1245 (1.5045)	0.9341
DL-PCB 105 (ng/L)	0.4170	1.8651 (1.7705)	0.2926	0.3307	−0.5484 (1.7947)	0.7601
DL-PCB 118 (ng/L)	0.4163	0.3552 (0.4085)	0.3850	0.3339	−0.3153 (0.3618)	0.3840
DL-PCB 156 (ng/L)	0.4141	−0.1520 (0.7529)	0.8401	0.3462	−1.5856 (0.6564)	0.0161
DL-PCB 157 (ng/L)	0.4242	−4.5409 (2.3586)	0.0548	0.3719	−7.1843 (1.6857)	<0.0001
DL-PCB 167 (ng/L)	0.4143	−0.9897 (3.2387)	0.7601	0.3499	−6.5369 (2.6521)	0.0140
DL-PCB 189 (ng/L)	0.4217	−7.7779 (5.0742)	0.1260	0.3673	−14.2291 (4.2063)	0.0008
ΣDL-PCBs^g^ (ng/L)	0.4142	0.0712 (0.2391)	0.7660	0.3422	−0.3521 (0.2110)	0.0958

a Model adjusted for age, total plasma lipids, menopausal status, education level, supplement use (calcium and vitamin D), blood mercury levels, blood lead levels, omega-3 PUFA, omega-6 PUFA, vitamin A (retinol), vitamin D, tobacco use, parity, height, weight

b model adjusted for age and total plasma lipids

c weighted and rounded size

d sample size

e the values of the regression coefficients and standard errors were multiplied by 10^4^

f Wald Chi square test with Satterthwaite correction for the degrees of freedom

g sum of DL-PCBs nos. 105, 118, 156, 157, 167 and 189

#### Factors significantly associated with SI (log)

In multivariate models adjusted for 15 confounders and covariates (model 1), 3 variables were found to be associated with SI. Age was negatively associated with SI in all models. Taking supplements (calcium and vitamin D) was negatively associated with SI in models where the main exposure variable was either total DLCs, DL-PCB 105, DL-PCB 118, DL-PCB 156, DL-PCB 167 or the sum of DL-PCBs. The omega-3 PUFA content of erythrocyte's membrane phospholipids was positively associated with SI in models comprising either total DLCs, DL-PCB 156, DL-PCB 167 or the sum of DL-PCBs as the main exposure variable. Generally speaking, SI (log) decreased with age and with supplement (calcium and vitamin D) use and SI values (log) increased with the content of omega-3 PUFAs in erythrocyte membrane phospholipids (data not shown).

## Discussion

We have investigated the relation between exposure to total DLCs or specific DL-PCBs and bone strength assessed by QUS in a representative sample of Inuit women from Nunavik. Neither the total DLC measure obtained using the reporter gene bioassay nor specific DL-PCBs congeners were associated with SI in multivariate models.

To the best of our knowledge, this is the first study to investigate the relationship between exposure to DLCs measured globally using an AhR-responsive cell-based reporter gene bioassay and bone quality, either assessed by QUS parameters or BMD. In contrast, 3 studies investigated exposure to DL-PCBs as a risk factor of lower bone quality and they yielded conflicting results.

In the study by Glynn et al. (23) conducted among Swedish men from the general population (n=115, aged 40–75 years), a positive association of borderline significance (β=0.0044, p=0.05) was found between DL-PCB 167 serum concentrations and total body BMD measured by dual-energy X-ray absorptiometry (DXA), after adjustment for several confounding factors. However, the authors found no relationship between DL-PCB 167 serum levels and QUS parameters (BUA and SOS) measured at the left calcaneus. Serum levels of DL-PCBs nos. 105, 118 and 156 were neither associated with QUS parameters nor with BMD measured at the spine, femoral neck or total body level (23).

In multivariate regression analyses, Côté et al. (13) observed that plasma DL-PCB 156 concentrations were negatively associated with QUS bone parameters in peri- and postmenopausal Inuit women from Nuuk, Greenland (SOS model: β=−22.68, p=0.014; BUA model: β=−8.12, p=0.028, SI model: β=−11.95, p=0.009). However, residual confounding may be present, because the models were only adjusted for 4 factors (age, weight, oral contraceptives and hormone replacement therapy). In the same study, DL-PCB 105 and DL-PCB 118 plasma levels were not associated with QUS parameters (13).

Hodgson et al. (24) identified in the multiple regression models a significant negative relationship (β=−0.00024, p=0.002) between serum DL-PCB 118 concentrations and BMD measured by DXA at the forearm (radius and ulna, non-dominant arm) of 154 Swedish men (60–81 years) participating in the OSCAR cohort. In contrast, among 167 women enrolled in the same study, DL-PCB 118 levels were positively associated with BMD (β=0.00008, p=0.045). No relationship between levels of other DL-PCBs (nos. 105, 156, 157 and 167) and BMD was observed. The authors pointed out problems such as an insufficient statistical power and a low proportion of the variance in BMD explained by the models (24).

Mean plasma concentrations of DLCs in Inuit women from Nunavik were lower than those reported in Inuit women from 6 regions of Greenland using a similar bioassay (38). Geometric mean (GM) concentrations of specific DL-PCB congeners in plasma samples of Inuit women were greater than those reported for women aged 40–79 years who participated in the Canadian Health Measures Survey in 2007–2009, and who were representative of the general Canadian population (39).

The mean SI value of 78.7% reported here for Nunavik women was lower than that of 91.5% measured using the same instrument in 249 Cree women of comparable age (mean age of 48 years) from East James Bay (Northern Quebec) (40).

Our study has several strengths. Firstly, the data used in this work were obtained from a large health survey conducted in the Inuit population of Nunavik, a unique population with a relatively high exposure to persistent organic pollutants. Our population sample was likely representative of all women aged 35–72 years of Nunavik, because of the study design, the recruitment strategy and the weighting scheme that takes into account non-response and refusals to participate. Secondly, we considered a large number of confounding factors and covariates, some of them investigated for the first time (omega-3 and omega-6 PUFAs, retinol, mercury). Thirdly, measurement biases on the dependent variable or exposure variables are unlikely. QUS parameters, anthropometric measures and biological sampling were all performed by research nurses using standardized techniques. DL-PCBs and total DLC analyses were performed at the *Laboratoire de Toxicologie* of the INSPQ, which is accredited under ISO 17025 by the Standards Council of Canada. QA/QC measures were all within acceptable ranges.

However, this study has some limitations. Firstly, the main methodological limitation is its cross-sectional design, with exposure and the dependent variable measured at the same time, such that the temporal sequence of cause and effect cannot be determined. However, due to the fact that DLCs and DL-PCBs accumulate in the body with age, plasma concentrations in Inuit women reflect their exposure throughout their lifetime. Secondly, the participation rate of women aged 35–72 years at QUS measurements was relatively small, which could potentially lead to a selection bias. However, this bias is quantitatively unimportant, since it is unlikely that the characteristics of the women included in the study would be different from those of all eligible women. Regarding the non-response and refusal to participate in the study, it is unlikely that the non-participants would have different levels in plasma DLCs, DL-PCB congeners or SI values than those of the participants; Inuit were recruited for a general health survey and not specifically for the purpose of our study on bone health. Thirdly, the number of participants was relatively small, limiting our capacity to observe an association between DLC concentrations determined by the reporter gene assay and QUS parameters, especially considering that the bioassay is relatively imprecise compared to analytical chemistry based data. Lastly, the reporter gene assay does not allow the identification of specific compounds involved in triggering the transcriptional response. The bioassay provides a global measure of activity for the complex mixture of persistent organic pollutants extracted from plasma samples (i.e. PCDDs, PCDFs and DL-PCBs). Other environmental contaminants such as brominated dioxins, polychlorinated terphenyls, polychlorinated napthalenes can also interact with the AhR-signalling pathway and may have contributed to the global response measured.

In conclusion, despite being exposed to DLCs through their traditional diet, we found no association between this exposure and bone strength, assessed by QUS, in Inuit women of Nunavik.
